# Supplementary material for: Modeling Latent Neural Dynamics with Gaussian Process Switching Linear Dynamical Systems
Source: ArXiv. 2025 Jan 13:arXiv:2408.03330v3. Preprint. [Version 3] (PMC11774443)
Supplement: Supplement 1 [file NIHPP2408.03330v3-supplement-1.pdf]

# Supplementary Material

## Table of Contents

– **Appendix A**: Relationship between linear kernel and linear dynamical systems
– **Appendix B**: Inference and learning
– **Appendix C**: Empirical results for new learning objective
– **Appendix D**: Additional synthetic data results

# A Relationship between linear kernel and linear dynamical systems

Here, we draw a mathematical connection between functions sampled from a GP with a linear kernel,

$$f(\cdot) \sim \mathcal{GP}(0, \kappa_{\text{lin}}(\cdot, \cdot)), \qquad \kappa_{\text{lin}}(\boldsymbol{x}, \boldsymbol{x}') = (\boldsymbol{x} - \boldsymbol{c})^\mathsf{T} \boldsymbol{M} (\boldsymbol{x} - \boldsymbol{c}) + \sigma_0^2 \tag{11}$$

and functions of the form

$$f(\boldsymbol{x}) = \boldsymbol{\beta}^\mathsf{T} \boldsymbol{x} + \beta_0. \tag{12}$$

This connection is useful for understanding the relationship between our model formulation and the typical linear dynamical systems formulation as in the rSLDS. In particular, we will show that the linear kernel in eq. (11) equivalently places a prior on $\boldsymbol{\beta}$ and $\beta_0$ in eq. (12), in a similar manner to Bayesian linear regression.

By definition of a GP, for any finite set of input locations $\{\boldsymbol{x}_i\}_{i=1}^N$, we have

$$\begin{bmatrix} f(\boldsymbol{x}_1) & f(\boldsymbol{x}_2) & \dots & f(\boldsymbol{x}_N) \end{bmatrix}^\mathsf{T} \sim \mathcal{N}(\boldsymbol{0}, \Phi) \tag{13}$$

where $\Phi_{ij} = \kappa_{\text{lin}}(\boldsymbol{x}_i, \boldsymbol{x}_j)$. Equivalently, for every pair $i, j = 1, \dots, N$,

$$\begin{aligned} \text{Cov}(f(\boldsymbol{x}_i), f(\boldsymbol{x}_j)) &= (\boldsymbol{x}_i - \boldsymbol{c})^\mathsf{T} \boldsymbol{M} (\boldsymbol{x}_j - \boldsymbol{c}) + \sigma_0^2 & (14) \\ &= \boldsymbol{x}_i^\mathsf{T} \boldsymbol{M} \boldsymbol{x}_j - \boldsymbol{c}^\mathsf{T} \boldsymbol{M} (\boldsymbol{x}_i + \boldsymbol{x}_j) + \boldsymbol{c}^\mathsf{T} \boldsymbol{c} + \sigma_0^2 & (15) \end{aligned}$$

Under eq. (12), treating $\boldsymbol{\beta}$ and $\beta_0$ as random, this would become

$$\text{Cov}(\boldsymbol{\beta}^\mathsf{T} \boldsymbol{x}_i + \beta_0, \boldsymbol{\beta}^\mathsf{T} \boldsymbol{x}_j + \beta_0) = \boldsymbol{x}_i^\mathsf{T} \text{Cov}(\boldsymbol{\beta}, \boldsymbol{\beta}) \boldsymbol{x}_j + \text{Cov}(\beta_0, \boldsymbol{\beta})(\boldsymbol{x}_i + \boldsymbol{x}_j) + \text{Var}(\beta_0) \tag{16}$$

Equation (15) and eq. (16) are equivalent and eq. (13) is satisfied if and only if

$$\begin{bmatrix} \boldsymbol{\beta} \\ \beta_0 \end{bmatrix} \sim \mathcal{N} \left( \boldsymbol{0}, \begin{bmatrix} \boldsymbol{M} & -\boldsymbol{M}\boldsymbol{c} \\ -\boldsymbol{c}^\mathsf{T} \boldsymbol{M} & \boldsymbol{c}^\mathsf{T} \boldsymbol{c} + \sigma_0^2 \end{bmatrix} \right) \tag{17}$$

This shows that a GP with a linear kernel is equivalent to a Bayesian linear regression model with a prior on coefficients of the form in eq. (17).

# B Inference and learning

We present the full inference and learning details for the gpSLDS with additive inputs,

$$d\boldsymbol{x} = (\boldsymbol{f}(\boldsymbol{x}) + \boldsymbol{B}\boldsymbol{v}(t))dt + \boldsymbol{\Sigma}^{\frac{1}{2}} d\boldsymbol{w}, \qquad \mathbb{E}[\boldsymbol{y}(t_i) \mid \boldsymbol{x}] = g\left(\boldsymbol{C}\boldsymbol{x}(t_i) + \boldsymbol{d}\right). \tag{18}$$

Our approach primarily follows that of Duncker et al. [10]. We use the following notation from Duncker et al. [10] throughout this section:

- $\langle h(\cdot) \rangle_{p(\cdot)}$ denotes the expectation of $h(\cdot)$ with respect to the distribution $p(\cdot)$.
- $\boldsymbol{K}_{xz} \in \mathbb{R}^{N_1 \times N_2}$ denotes the covariance matrix defined by our kernel $\kappa_{\text{ssl}}(\cdot, \cdot)$ for two batches of points $\{\boldsymbol{x}_i\}_{i=1}^{N_1}$ and $\{\boldsymbol{z}_i\}_{i=1}^{N_2}$. Specifically, $[\boldsymbol{K}_{xz}]_{ij} = \kappa_{\text{ssl}}(\boldsymbol{x}_i, \boldsymbol{z}_j)$. If one of these batches has only one point, e.g. $N_1 = 1$, we denote the corresponding covariance vector as $\boldsymbol{k}_{xz} \in \mathbb{R}^{1 \times N_2}$. Note, these quantities depend on $\Theta$ through the kernel; we omit writing this dependence for brevity.

## B.1 Augmenting the generative model

Following Duncker et al. [10], we first augment our generative model with sparse inducing points $\{\boldsymbol{u}_k\}_{k=1}^K \subset \mathbb{R}^M$ at input locations $\{\boldsymbol{z}_m\}_{m=1}^M \subset \mathbb{R}^D$. Inducing points can be seen as pseudo-observations of $\boldsymbol{f}(\cdot)$ at input locations $\{\boldsymbol{z}_m\}_{m=1}^M$. They allow for tractable inference of $\boldsymbol{f}$ at any new batch of $N$ input locations by reducing computational complexity from $O(N^3)$ to $O(NM^2)$, where typically $M$ is chosen to be much smaller than $N$ [37]. After this augmentation, the joint likelihood of our model becomes

$$p(\boldsymbol{y}, \boldsymbol{x}, \boldsymbol{f}, \boldsymbol{u} \mid \Theta) = p(\boldsymbol{y} \mid \boldsymbol{x}) p(\boldsymbol{x} \mid \boldsymbol{f}) \prod_{k=1}^K p(f_k \mid \boldsymbol{u}_k, \Theta) p(\boldsymbol{u}_k \mid \Theta). \tag{19}$$

Treating $\boldsymbol{u}_k$ as pseudo-observations, we assume the following augmented prior:

$$p(\boldsymbol{u}_k \mid \Theta) = \mathcal{N}(\boldsymbol{u}_k \mid \mathbf{0}, \boldsymbol{K}_{zz}). \tag{20}$$

Then we can view $f_k(\cdot) \mid \boldsymbol{u}_k$ as a new GP conditioned on $\boldsymbol{u}_k$,

$$f_k(\cdot) \mid \boldsymbol{u}_k \sim \mathcal{GP}(\mu_{f_k|\boldsymbol{u}_k}(\cdot), \kappa_{f_k|\boldsymbol{u}_k}(\cdot,\cdot)), \tag{21}$$

where

$$\mu_{f_k|\boldsymbol{u}_k}(\boldsymbol{x}) = \boldsymbol{k}_{xz}\boldsymbol{K}_{zz}^{-1}\boldsymbol{u}_k$$
$$\kappa_{f_k|\boldsymbol{u}_k}(\boldsymbol{x},\boldsymbol{x}') = \kappa_{\text{ssl}}(\boldsymbol{x},\boldsymbol{x}') - \boldsymbol{k}_{xz}\boldsymbol{K}_{zz}^{-1}\boldsymbol{k}_{zx}.$$

### B.2 Variational lower bound

As in Duncker et al. [10], we consider a variational approximation to the posterior of the form

$$q(\boldsymbol{x}, \boldsymbol{f}, \boldsymbol{u}) = q(\boldsymbol{x}) \prod_{k=1}^{K} p(f_k \mid \boldsymbol{u}_k, \Theta) q(\boldsymbol{u}_k). \tag{22}$$

Using this factorization, we derive the ELBO to the marginal log-likelihood of our model. By Jensen's inequality,

$$\begin{aligned}
\log p(\boldsymbol{y} \mid \Theta) &= \log \int p(\boldsymbol{y} \mid \boldsymbol{x}) p(\boldsymbol{x} \mid \boldsymbol{f}) p(\boldsymbol{f} \mid \boldsymbol{u}, \Theta) p(\boldsymbol{u} \mid \Theta) d\boldsymbol{x} d\boldsymbol{f} d\boldsymbol{u} \\
&\geq \int q(\boldsymbol{x}, \boldsymbol{f}, \boldsymbol{u}) \log \frac{p(\boldsymbol{y} \mid \boldsymbol{x}) p(\boldsymbol{x} \mid \boldsymbol{f}) p(\boldsymbol{f} \mid \boldsymbol{u}, \Theta) p(\boldsymbol{u} \mid \Theta)}{q(\boldsymbol{x}, \boldsymbol{f}, \boldsymbol{u})} d\boldsymbol{x} d\boldsymbol{f} d\boldsymbol{u} \\
&= \int q(\boldsymbol{x}, \boldsymbol{f}, \boldsymbol{u}) \log \frac{p(\boldsymbol{y} \mid \boldsymbol{x}) p(\boldsymbol{x} \mid \boldsymbol{f}) \prod_{k=1}^{K} p(\boldsymbol{u}_k \mid \Theta)}{q(\boldsymbol{x}) \prod_{k=1}^{K} q(\boldsymbol{u}_k)} d\boldsymbol{x} d\boldsymbol{f} d\boldsymbol{u} \\
&= \langle \log p(\boldsymbol{y} \mid \boldsymbol{x}) \rangle_{q(\boldsymbol{x})} - \langle \mathrm{KL}[q(\boldsymbol{x}) || p(\boldsymbol{x} \mid \boldsymbol{f})] \rangle_{q(\boldsymbol{f})} - \sum_{k=1}^{K} \mathrm{KL}[q(\boldsymbol{u}_k) || p(\boldsymbol{u}_k \mid \Theta)] \\
&:= \mathcal{L}(q(\boldsymbol{x}), q(\boldsymbol{u}), \Theta),
\end{aligned}$$

where

$$q(\boldsymbol{f}) = \prod_{k=1}^{K} \int p(f_k \mid \boldsymbol{u}_k, \Theta) q(\boldsymbol{u}_k) d\boldsymbol{u}_k. \tag{23}$$

### B.3 Inference of latent paths

To perform inference over the posterior of latent paths $q(\boldsymbol{x})$, we follow a method first proposed in Archambeau et al. [39] and extended by Duncker et al. [10].

As in Archambeau et al. [39], we choose a posterior distribution $q(\boldsymbol{x})$ characterized by a Markov Gaussian process,

$$q(\boldsymbol{x}) : \{d\boldsymbol{x} = \underbrace{(-\boldsymbol{A}(t)\boldsymbol{x}(t) + \boldsymbol{b}(t))}_{:=\boldsymbol{f}_q(\boldsymbol{x})} dt + \boldsymbol{\Sigma}^{\frac{1}{2}} d\boldsymbol{w}, \quad \boldsymbol{x}_0 \sim \mathcal{N}(\boldsymbol{m}(0), \boldsymbol{S}(0))\}. \tag{24}$$

This distribution satisfies posterior marginals $q(\boldsymbol{x}_t) = \mathcal{N}(\boldsymbol{x}_t \mid \boldsymbol{m}_t, \boldsymbol{S}_t)$ which satisfy the differential equations

$$\frac{d\boldsymbol{m}(t)}{dt} = -\boldsymbol{A}(t)\boldsymbol{m}(t) + \boldsymbol{b}(t) \tag{25}$$
$$\frac{d\boldsymbol{S}(t)}{dt} = -\boldsymbol{A}(t)\boldsymbol{S}(t) - \boldsymbol{S}(t)\boldsymbol{A}(t)^{\mathsf{T}} + \boldsymbol{\Sigma}. \tag{26}$$

Archambeau et al. [39] maximize the ELBO with respect to $q(\boldsymbol{x})$ subject to the constraints in eq. (25) and eq. (26) using the method of Lagrange multipliers. They show that after applying integration by parts, the Lagrangian becomes

$$\widetilde{\mathcal{L}} = \mathcal{L}(q(\boldsymbol{x}), q(\boldsymbol{u}), \Theta) - \mathcal{C}_1 - \mathcal{C}_2 \tag{27}$$

where

$$\mathcal{C}_1 = \int_0^T \left\{ \boldsymbol{\lambda}(t)^\top (\boldsymbol{A}(t)\boldsymbol{m}(t) - \boldsymbol{b}(t)) - \frac{d\boldsymbol{\lambda}(t)}{dt}^\top \boldsymbol{m}(t) \right\} dt + \boldsymbol{\lambda}(T)^\top \boldsymbol{m}(T) - \boldsymbol{\lambda}(0)^\top \boldsymbol{m}(0) \tag{28}$$

$$\mathcal{C}_2 = \int_0^T \mathrm{Tr}\left[ \boldsymbol{\Psi}(t)(\boldsymbol{A}(t)\boldsymbol{S}(t) + \boldsymbol{S}(t)\boldsymbol{A}(t)^\top - \boldsymbol{\Sigma}) - \frac{d\boldsymbol{\Psi}(t)}{dt}\boldsymbol{S}(t) \right] dt \tag{29}$$

$$+ \mathrm{Tr}[\boldsymbol{\Psi}(T)\boldsymbol{S}(T)] - \mathrm{Tr}[\boldsymbol{\Psi}(0)\boldsymbol{S}(0)] \tag{30}$$

As in Archambeau et al. [39], we assume that $\boldsymbol{\lambda}(T) = \mathbf{0}$ and $\boldsymbol{\Psi}(T) = \mathbf{0}$.

To find the stationary points of the Lagrangian, we first take derivatives of $\widetilde{\mathcal{L}}$ with respect to $\boldsymbol{m}(0), \boldsymbol{S}(0), \boldsymbol{m}(t), \boldsymbol{S}(t), \boldsymbol{A}(t), \boldsymbol{b}(t)$ and set them to 0. The derivatives with respect to $\boldsymbol{m}(0)$ and $\boldsymbol{S}(0)$ lead to the updates

$$\boldsymbol{m}(0) = \boldsymbol{\mu}(0) - \boldsymbol{V}(0)\boldsymbol{\lambda}(0), \qquad \boldsymbol{S}(0) = \left(2\boldsymbol{\Psi}(0) + \boldsymbol{V}(0)^{-1}\right)^{-1} \tag{31}$$

where we assume a prior on initial conditions $p(\boldsymbol{x}_0) = \mathcal{N}(\boldsymbol{x}_0 \mid \boldsymbol{\mu}(0), \boldsymbol{V}(0))$.

The derivatives with respect to $\boldsymbol{m}(t)$ and $\boldsymbol{S}(t)$ lead to the stationary equations

$$\frac{d\boldsymbol{\lambda}(t)}{dt} = \boldsymbol{A}(t)^\top \boldsymbol{\lambda}(t) - \frac{\partial \mathcal{L}}{\partial \boldsymbol{m}(t)} \tag{32}$$

$$\frac{d\boldsymbol{\Psi}(t)}{dt} = \boldsymbol{A}(t)^\top \boldsymbol{\Psi}(t) - \boldsymbol{\Psi}(t)\boldsymbol{A}(t) - \frac{\partial \mathcal{L}}{\partial \boldsymbol{S}(t)} \odot \mathbb{P} \tag{33}$$

with $\mathbb{P}_{ij} = \frac{1}{2}$ for $i \neq j$ and 1 otherwise. The inclusion of $\mathbb{P}$ was proposed by Duncker et al. [10] to adjust for taking derivatives with respect to a symmetric matrix.

To take derivatives with respect to $\boldsymbol{A}(t)$ and $\boldsymbol{b}(t)$, we first extend a result from Appendix A of Archambeau et al. [39] to our affine inputs model. This allows us to rewrite the KL-divergence term between the posterior and prior latent paths in the ELBO as

$$\begin{aligned}
\langle \mathrm{KL}[q(\boldsymbol{x}) || p(\boldsymbol{x} \mid \boldsymbol{f})] \rangle_{q(\boldsymbol{f})} &= \frac{1}{2}\int_0^T \left\langle (\boldsymbol{f} + \boldsymbol{B}\boldsymbol{v}(t) - \boldsymbol{f}_q)^\top (\boldsymbol{f} + \boldsymbol{B}\boldsymbol{v}(t) - \boldsymbol{f}_q) \right\rangle_{q(\boldsymbol{x}),q(\boldsymbol{f})} dt \\
&= \frac{1}{2}\int_0^T \left\langle (\boldsymbol{B}\boldsymbol{v}(t) + \Delta\boldsymbol{f})^\top (\boldsymbol{B}\boldsymbol{v}(t) + \Delta\boldsymbol{f}) \right\rangle_{q(\boldsymbol{x}),q(\boldsymbol{f})} dt \\
&= \frac{1}{2}\int_0^T \left\langle (\Delta\boldsymbol{f})^\top (\Delta\boldsymbol{f}) \right\rangle_{q(\boldsymbol{x}),q(\boldsymbol{f})} dt \\
&\quad + \int_0^T \boldsymbol{v}(t)^\top \boldsymbol{B}^\top \langle \Delta\boldsymbol{f} \rangle_{q(\boldsymbol{x}),q(\boldsymbol{f})} dt + \frac{1}{2}\int_0^T \boldsymbol{v}(t)^\top \boldsymbol{B}^\top \boldsymbol{B}\boldsymbol{v}(t) dt
\end{aligned} \tag{34}$$

where $\Delta\boldsymbol{f} := \boldsymbol{f} - \boldsymbol{f}_q$. The integrand of the first term in eq. (34) can be expanded as

$$\begin{aligned}
\left\langle (\Delta\boldsymbol{f})^\top (\Delta\boldsymbol{f}) \right\rangle_{q(\boldsymbol{x}),q(\boldsymbol{f})} &= \left\langle \boldsymbol{f}^\top \boldsymbol{f} \right\rangle_{q(\boldsymbol{x}),q(\boldsymbol{f})} + 2\mathrm{Tr}\left[ \boldsymbol{A}(t)^\top \left\langle \frac{\partial \boldsymbol{f}}{\partial \boldsymbol{x}} \right\rangle_{q(\boldsymbol{x}),q(\boldsymbol{f})} \boldsymbol{S}(t) \right] \\
&\quad + \mathrm{Tr}\left[ \boldsymbol{A}(t)^\top \boldsymbol{A}(t)(\boldsymbol{S}(t) + \boldsymbol{m}(t)\boldsymbol{m}(t)^\top) \right] + 2\boldsymbol{m}(t)^\top \boldsymbol{A}(t)^\top \langle \boldsymbol{f} \rangle_{q(\boldsymbol{x}),q(\boldsymbol{f})} \\
&\quad + \boldsymbol{b}(t)^\top \boldsymbol{b}(t) - 2\boldsymbol{b}(t)^\top \langle \boldsymbol{f} \rangle_{q(\boldsymbol{x}),q(\boldsymbol{f})} - 2\boldsymbol{b}^\top \boldsymbol{A}(t)\boldsymbol{m}(t)
\end{aligned} \tag{35}$$

where we have used the identity $\left\langle \langle \boldsymbol{f}(\boldsymbol{x}) \rangle_{q(\boldsymbol{f})} (\boldsymbol{x} - \boldsymbol{m})^\top \right\rangle_{q(\boldsymbol{x})} = \left\langle \frac{\partial \boldsymbol{f}}{\partial \boldsymbol{x}} \right\rangle_{q(\boldsymbol{x}),q(\boldsymbol{f})} \boldsymbol{S}$, which can be derived from Stein's lemma. Note that computing eq. (35) relies on three quantities,

$$\langle \boldsymbol{f} \rangle_{q(\boldsymbol{x}),q(\boldsymbol{f})}, \left\langle \boldsymbol{f}^\top \boldsymbol{f} \right\rangle_{q(\boldsymbol{x}),q(\boldsymbol{f})}, \left\langle \frac{\partial \boldsymbol{f}}{\partial \boldsymbol{x}} \right\rangle_{q(\boldsymbol{x}),q(\boldsymbol{f})}$$

which can be written as terms which depend on expectations of the kernel with respect to $q(\boldsymbol{x})$. We derive these as follows:

$$\begin{aligned}
\langle \boldsymbol{f} \rangle_{q(\boldsymbol{x}),q(\boldsymbol{f})} &= \left\langle \boldsymbol{k}_{xz} \boldsymbol{K}_{zz}^{-1} \boldsymbol{u} \right\rangle_{q(\boldsymbol{u}),q(\boldsymbol{x})} \\
&= \langle \boldsymbol{k}_{xz} \rangle_{q(\boldsymbol{x})} \boldsymbol{K}_{zz}^{-1} \boldsymbol{m}_u
\end{aligned}$$

$$
\begin{aligned}
\left\langle \boldsymbol{f}^\mathsf{T}\boldsymbol{f}\right\rangle_{q(\boldsymbol{x}),q(\boldsymbol{f})} &= \left\langle \sum_{k=1}^{K} \left\langle f_k(\boldsymbol{x})^2 \right\rangle_{q(f_k)} \right\rangle_{q(\boldsymbol{x})} \\
&= \left\langle \sum_{k=1}^{K} \mathrm{Var}_{q(f_k)}[f_k(\boldsymbol{x})] + \left\langle f_k(\boldsymbol{x})\right\rangle^2_{q(f_k)} \right\rangle_{q(\boldsymbol{x})} \\
&= \sum_{k=1}^{K} \underbrace{\left\langle \mathrm{Var}_{p(f_k|\boldsymbol{u})}[f_k(\boldsymbol{x})]\right\rangle_{q(\boldsymbol{x}),q(\boldsymbol{u})}}_{\text{Term 1}} + \underbrace{\left\langle \mathrm{Var}_{q(u)}[\langle f_k(\boldsymbol{x})\rangle_{p(f_k \mid \boldsymbol{u})}]\right\rangle_{q(\boldsymbol{x})}}_{\text{Term 2}} \\
&\quad + \underbrace{\left\langle \langle f_k(\boldsymbol{x})\rangle^2_{p(f_k \mid \boldsymbol{u}),q(\boldsymbol{u})}\right\rangle_{q(\boldsymbol{x})}}_{\text{Term 3}} \\
&= \sum_{k=1}^{K} \underbrace{\left\langle \boldsymbol{k}_{xx} - \boldsymbol{k}_{xz}\boldsymbol{K}_{zz}^{-1}\boldsymbol{k}_{zx}\right\rangle_{q(\boldsymbol{x})}}_{\text{Term 1}} + \underbrace{\left\langle \boldsymbol{k}_{xz}\boldsymbol{K}_{zz}^{-1}\boldsymbol{S}_u^k\boldsymbol{K}_{zz}^{-1}\boldsymbol{k}_{zx}\right\rangle_{q(\boldsymbol{x})}}_{\text{Term 2}} \\
&\quad + \underbrace{\left\langle \boldsymbol{k}_{xz}\boldsymbol{K}_{zz}^{-1}\boldsymbol{m}_u^k(\boldsymbol{m}_u^k)^T\boldsymbol{K}_{zz}^{-1}\boldsymbol{k}_{zx}\right\rangle_{q(\boldsymbol{x})}}_{\text{Term 3}}
\end{aligned}
$$

$$
\begin{aligned}
\left\langle \frac{\partial \boldsymbol{f}}{\partial \boldsymbol{x}} \right\rangle_{q(\boldsymbol{x}),q(\boldsymbol{f})} &= \left\langle \boldsymbol{m}_u^\mathsf{T}\boldsymbol{K}_{zz}^{-1}\tfrac{\partial \boldsymbol{k}_{zx}}{d\boldsymbol{x}}\right\rangle_{q(\boldsymbol{x})} \\
&= \boldsymbol{m}_u^\mathsf{T}\boldsymbol{K}_{zz}^{-1}\left\langle \tfrac{\partial \boldsymbol{k}_{zx}}{d\boldsymbol{x}}\right\rangle_{q(\boldsymbol{x})}
\end{aligned}
$$

The above three function expectations can thus be expressed in terms of the following four kernel expectations with respect to $q(\boldsymbol{x}) = \mathcal{N}(\boldsymbol{x} \mid \boldsymbol{m}, \boldsymbol{S})$:

$$
\langle \kappa(\boldsymbol{x},\boldsymbol{x})\rangle_{q(\boldsymbol{x})}, \quad \langle \kappa(\boldsymbol{x},\boldsymbol{z})\rangle_{q(\boldsymbol{x})}, \quad \langle \kappa(\boldsymbol{z}_1,\boldsymbol{x})\kappa(\boldsymbol{x},\boldsymbol{z}_2)\rangle_{q(\boldsymbol{x})}, \quad \left\langle \frac{\partial \kappa(\boldsymbol{z},\boldsymbol{x})}{\partial \boldsymbol{x}}\right\rangle_{q(\boldsymbol{x})}.
$$

For our SSL kernel these kernel expectations are not available in closed form, so in practice we approximate them using Gauss-Hermite quadrature.

Next, differentiating $\widetilde{\mathcal{L}}$ with respect to $\boldsymbol{A}(t)$ and $\boldsymbol{b}(t)$ yields the updates

$$
\boldsymbol{A}(t) = -\left\langle \frac{\partial \boldsymbol{f}}{\partial \boldsymbol{x}}\right\rangle_{q(\boldsymbol{x}),q(\boldsymbol{f})} + 2\boldsymbol{\Sigma}\boldsymbol{\Psi}(t) \tag{36}
$$

$$
\boldsymbol{b}(t) = \langle \boldsymbol{f}(\boldsymbol{x})\rangle_{q(\boldsymbol{x}),q(\boldsymbol{f})} + \boldsymbol{A}(t)\boldsymbol{m}(t) + \boldsymbol{B}\boldsymbol{v}(t) - \boldsymbol{\Sigma}\boldsymbol{\lambda}(t) \tag{37}
$$

Note that these updates have one key difference from the previously derived updates in Archambeau et al. [39] and Duncker et al. [10]: the input-dependent term $\boldsymbol{B}\boldsymbol{v}(t)$ in eq. (37). Intuitively, this is because the posterior bias term $\boldsymbol{b}(t)$ is fully time-varying, so it captures changes in the latent states due to input-driven effects in the posterior.

In summary, the inference algorithm for updating $q(\boldsymbol{x})$ is as follows. In each iteration of vEM, we repeat the following forward-backward style algorithm:

1. Solve for $\boldsymbol{m}(t)$, $\boldsymbol{S}(t)$ forward in time starting from $\boldsymbol{m}(0)$, $\boldsymbol{S}(0)$ via eq. (25) and eq. (26).
2. Solve for $\boldsymbol{\lambda}(t)$, $\boldsymbol{\Psi}(t)$ backward in time starting from $\boldsymbol{\lambda}(T), \boldsymbol{\Psi}(T) = \boldsymbol{0}$ via eq. (32) and eq. (33).
3. Update $\boldsymbol{A}(t)$ and $\boldsymbol{b}(t)$ via eq. (36) and eq. (37).

After solving these stationary equations, we update $\boldsymbol{m}(0)$ and $\boldsymbol{S}(0)$ via eq. (31).

**Computational details** Solving for $\boldsymbol{m}(t)$, $\boldsymbol{S}(t)$, $\boldsymbol{\lambda}(t)$, and $\boldsymbol{\Psi}(t)$ requires integrating continuous-time ODEs. In practice we use Euler integration with a small discretization step $\Delta t$ relative to the sampling rate of the data, though in principle any ODE solver can be used. We found that the ELBO usually converges within 20 forward-backward iterations.

The ODEs for solving $\boldsymbol{\lambda}(t)$ and $\boldsymbol{\Psi}(t)$ in eq. (32) and eq. (33) depend on evaluating gradients of the ELBO with respect to $\boldsymbol{m}(t)$ and $\boldsymbol{S}(t)$. We use modern autodifferentiation capabilities in JAX to compute these gradients.

### B.4 Updating dynamics and hyperparameters with a modified learning objective

As we describe in Section 3.3, our gpSLDS inference algorithm uses a modified objective for hyperparameter learning. In this section, we discuss this objective in detail and present closed-form updates for the inducing points given the hyperparameters. Then, using the inducing points, we will derive the posterior distribution over $\boldsymbol{f}(\cdot)$ at any location in the latent space.

After updating the latent paths $q(\boldsymbol{x})$ as described in Appendix B.3, we update hyperparameters $\Theta$ using a partially optimized ELBO. This update can be written as

$$\Theta^* = \arg\max_{\Theta} \left\{ \max_{q(\boldsymbol{u})} \mathcal{L}(q(\boldsymbol{x}), q(\boldsymbol{u}), \Theta) \right\}. \tag{38}$$

Following Duncker et al. [10], we choose the variational posterior

$$q(\boldsymbol{u}_k) = \mathcal{N}(\boldsymbol{u}_k \mid \boldsymbol{m}_u^k, \boldsymbol{S}_u^k). \tag{39}$$

Given $q(\boldsymbol{x})$ and $\Theta$, this leads to the closed-form updates,

$$\boldsymbol{S}_u^{k*} = \boldsymbol{K}_{zz} \left( \boldsymbol{K}_{zz} + \int_0^T \langle \boldsymbol{k}_{zx} \boldsymbol{k}_{xz} \rangle_{q(\boldsymbol{x})} \, dt \right)^{-1} \boldsymbol{K}_{zz} \tag{40}$$

$$\begin{aligned} \boldsymbol{m}_u^* = \boldsymbol{S}_u^{k*} \boldsymbol{K}_{zz}^{-1} \int_0^T & \Big( \langle \boldsymbol{k}_{zx} \rangle_{q(\boldsymbol{x})} (-\boldsymbol{A}(t)\boldsymbol{m}(t) + \boldsymbol{b}(t) - \boldsymbol{B}\boldsymbol{v}(t))^\mathsf{T} \\ & - \left\langle \frac{\partial \boldsymbol{k}_{zx}}{\partial \boldsymbol{x}} \right\rangle_{q(\boldsymbol{x})} \boldsymbol{S}(t) \boldsymbol{A}(t)^\mathsf{T} \Big) \, dt \end{aligned} \tag{41}$$

In the above equation, $\boldsymbol{m}_u^* \in \mathbb{R}^{M \times K}$ contains $\boldsymbol{m}_u^{k*} \in \mathbb{R}^M$ in each column. The inside maximization of eq. (38) can be computed analytically using these closed-form updates. Note that $\boldsymbol{m}_u^{k*}$ and $\boldsymbol{S}_u^{k*}$ depend on $\Theta$ through the prior kernel covariances $\boldsymbol{K}_{zz}$ and $\boldsymbol{k}_{zx}$. Therefore, eq. (38) can be understood as performing joint optimization of the ELBO with respect to $\Theta$ through $\boldsymbol{m}_u^{k*}$ and $\boldsymbol{S}_u^{k*}$, as well as through the rest of the ELBO. In practice, this allows vEM to circumvent dependencies between $q(\boldsymbol{u})$ and $\Theta$, leading to more accurate estimation of both quantities. For our experiments, we use the Adam optimizer to solve eq. (38).

After obtaining $\Theta^*$ in each vEM iteration, we explicitly update $q(\boldsymbol{u})$ using eq. (41) and eq. (40) for the next iteration.

**Recovering predicted dynamics** Given (updated) variational parameters $\boldsymbol{m}_u^k$ and $\boldsymbol{S}_u^k$, it is straightforward to compute the posterior distribution of $\boldsymbol{f}^* := \boldsymbol{f}(\boldsymbol{x}^*)$ at any location $\boldsymbol{x}^*$ in the latent space. Recall the variational approximation from eq. (23). If we apply this approximation to $\boldsymbol{f}^*$, we have

$$q(\boldsymbol{f}^*) = \prod_{k=1}^K q(f_k^*) = \prod_{k=1}^K \int p(f_k^* \mid \boldsymbol{u}_k, \Theta) q(\boldsymbol{u}_k) d\boldsymbol{u}_k. \tag{42}$$

To evaluate this analytically, we use properties of conditional Gaussian distributions. First note that by our augmented prior,

$$p(f_k^* \mid \boldsymbol{u}_k, \Theta) = \mathcal{N}(f_k^* \mid \boldsymbol{k}_{x^*z} \boldsymbol{K}_{zz}^{-1} \boldsymbol{u}_k, \kappa_{\text{ssl}}(\boldsymbol{x}^*, \boldsymbol{x}^*) - \boldsymbol{k}_{x^*z} \boldsymbol{K}_{zz}^{-1} \boldsymbol{k}_{zx^*}). \tag{43}$$

Then, by conjugacy of Gaussian distributions,

$$\begin{aligned} q(f_k^*) &= \int \mathcal{N}(f_k^* \mid \boldsymbol{k}_{x^*z} \boldsymbol{K}_{zz}^{-1} \boldsymbol{u}_k, \kappa_{\text{ssl}}(\boldsymbol{x}^*, \boldsymbol{x}^*) - \boldsymbol{k}_{x^*z} \boldsymbol{K}_{zz}^{-1} \boldsymbol{k}_{zx^*}) \mathcal{N}(\boldsymbol{u}_k \mid \boldsymbol{m}_u^k, \boldsymbol{S}_u^k) d\boldsymbol{u}_k \\ &= \mathcal{N}(f_k^* \mid \boldsymbol{k}_{x^*z} \boldsymbol{K}_{zz}^{-1} \boldsymbol{m}_u^k, \kappa_{\text{ssl}}(\boldsymbol{x}^*, \boldsymbol{x}^*) - \boldsymbol{k}_{x^*z} \boldsymbol{K}_{zz}^{-1} \boldsymbol{k}_{zx^*} + \boldsymbol{k}_{x^*z} \boldsymbol{K}_{zz}^{-1} \boldsymbol{S}_u^k \boldsymbol{K}_{zz}^{-1} \boldsymbol{k}_{zx^*}). \end{aligned} \tag{44}$$

### B.5 Learning observation model parameters

For the experiments in this paper, we considered two observation models: Gaussian observations and Poisson process observations.

**Gaussian observations** We consider the observation model

$$p(\boldsymbol{y} \mid \boldsymbol{x}) = \prod_{t_i} \mathcal{N}(\boldsymbol{y}(t_i) \mid \boldsymbol{C}\boldsymbol{x}(t_i) + \boldsymbol{d}, \boldsymbol{R}). \tag{45}$$

where $\boldsymbol{R} \in \mathbb{R}^D$ is a diagonal covariance matrix. The expected log-likelihood is available in closed form and is given by

$$\langle \log p(\boldsymbol{y} \mid \boldsymbol{x}) \rangle_{q(\boldsymbol{x})} = \sum_{t_i} \left( \log \mathcal{N}(\boldsymbol{y}(t_i) \mid \boldsymbol{C}(t_i) + \boldsymbol{d}, \boldsymbol{R}) - \frac{1}{2} \mathrm{Tr} \left[ \boldsymbol{S}(t_i) \boldsymbol{C}^\top \boldsymbol{R}^{-1} \boldsymbol{C} \right] \right) \tag{46}$$

Closed-form updates for $\boldsymbol{C}, \boldsymbol{d}$ and $\boldsymbol{R}$ are also available:

$$\boldsymbol{C}^* = \left( \sum_{t_i} (\boldsymbol{y}(t_i) - \boldsymbol{d}) \boldsymbol{m}(t_i)^\top \right) \left( \sum_{t_i} (\boldsymbol{S}(t_i) + \boldsymbol{m}(t_i)\boldsymbol{m}(t_i)^\top) \right)^{-1} \tag{47}$$

$$\boldsymbol{d}^* = \frac{1}{n_{t_i}} \sum_{t_i} (\boldsymbol{y}(t_i) - \boldsymbol{C}^* \boldsymbol{m}(t_i)) \tag{48}$$

$$R_d^* = \frac{1}{n_{t_i}} \sum_{t_i} (y_d(t_i)^2 - 2y_d(t_i)\boldsymbol{c}_d^\top \boldsymbol{m}(t_i) + (\boldsymbol{c}_d^\top \boldsymbol{m}(t_i))^2 + \boldsymbol{c}_d^\top \boldsymbol{S}(t_i) \boldsymbol{c}_d) \tag{49}$$

where $n_{t_i}$ is the number of observed time points, $R_d^*$ is the $d$-th entry of $\boldsymbol{R}$, and $\boldsymbol{c}_d$ is the $d$-th row of $\boldsymbol{C}$.

**Poisson process observations** The second observation model we consider is Poisson process observations of the form

$$p(\{t_i\} \mid \boldsymbol{x}) = \mathcal{PP}(g(\boldsymbol{C}\boldsymbol{x}(t) + \boldsymbol{d})), \tag{50}$$

where either $g(a) = \exp(a)$ (exponential inverse link) or $g(a) = \log(1 + \exp(a))$ (softplus inverse link). For the exponential inverse link, the expected log-likelihood is available in closed form and is given by

$$\langle \log p(\{t_i\} \mid \boldsymbol{x}) \rangle_{q(\boldsymbol{x})} = -\int_0^T \exp\left( \boldsymbol{C}\boldsymbol{m}(t) + \boldsymbol{d} + \frac{1}{2} \mathrm{diag}(\boldsymbol{C}\boldsymbol{S}(t)\boldsymbol{C}^\top) \right) dt + \sum_{t_i} (\boldsymbol{C}\boldsymbol{m}(t_i) + \boldsymbol{d}) \tag{51}$$

For the softplus inverse link, the expected log-likelihood is not available in closed-form, but can be approximated by Gauss-Hermite quadrature or a second-order Taylor expansion around $\boldsymbol{m}(t)$.

For both Poisson process models, we update $\boldsymbol{C}$ and $\boldsymbol{d}$ using gradient ascent on the expected log-likelihood with the Adam optimizer.

### B.6 Learning the input effect matrix

Here we derive a closed-form update for $\boldsymbol{B}$, which linearly maps external inputs to the latent space. The only term in the ELBO which depends on $\boldsymbol{B}$ is $\langle \mathrm{KL}[q(\boldsymbol{x}) || p(\boldsymbol{x} \mid \boldsymbol{f})] \rangle_{q(\boldsymbol{f})}$. We differentiate this term as written in eq. (34) and arrive at the update

$$\boldsymbol{B}^* = -\left( \int_0^T (\langle \boldsymbol{f} \rangle_{q(\boldsymbol{x}), q(\boldsymbol{f})} + \boldsymbol{A}(t)\boldsymbol{m}(t) - \boldsymbol{b}(t)) \boldsymbol{v}(t)^\top dt \right) \left( \int_0^T \boldsymbol{v}(t)\boldsymbol{v}(t)^\top dt \right)^{-1}. \tag{52}$$

Note that the term $\left( \int_0^T \boldsymbol{v}(t)\boldsymbol{v}(t)^\top dt \right)^{-1}$ can be pre-computed since $\boldsymbol{v}(t)$ is known.

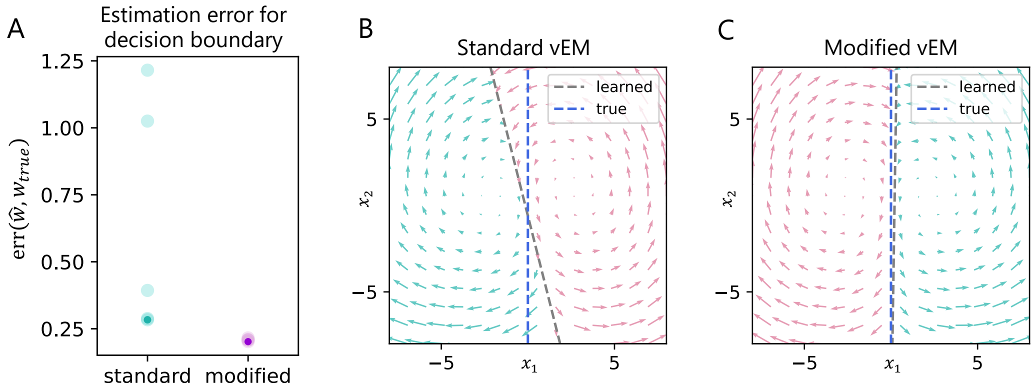


Figure 5: Comparison between the standard vEM approach in Duncker et al. [10] and our modified vEM approach. **A.** Estimation error between the true and learned decision boundaries, computed as described in Appendix C. For each vEM approach, we fit 5 gpSLDS models with different random initializations. The estimation errors are denoted in light blue/purple dots. The runs that we display in the next two panels are denoted by a solid blue/purple dot. **B.** The standard vEM approach fails to learn the true decision boundary of $x_1 = 0$. **C.** The modified vEM approach precisely learns this decision boundary.

# C Empirical results for new learning objective

In this section, we empirically compare the standard vEM approach from Duncker et al. [10] to our modified vEM approach in which we learn kernel hyperparameters on a partially optimized ELBO. For this experiment, we use the same synthetic dataset from our main result in Section 4.1. We fit the gpSLDS with 5 different random initializations using both standard vEM and modified vEM. For these fits, we fix the values of $\boldsymbol{C}$ and $\boldsymbol{d}$ throughout learning to ensure that the resulting models are anchored to the same latent subspace (in general, they are not guaranteed to end up in the same subspace due to rotational unidentifiability). Each run was fit with 50 total vEM iterations; each iteration consisted of 15 forward-backward solves to update $q(\boldsymbol{x})$ and 300 Adam gradient steps with a learning rate of 0.01 to update kernel hyperparameters.

To compare the quality of the learned hyperparameters, we quantitatively assess the error between the learned and true decision boundaries. In this simple example with $J = 2$, the decision boundary can be parametrized as $w_0 + w_1 x_1 + w_2 x_2 = 0$ for some $\boldsymbol{w} = (w_0, w_1, w_2)^\mathsf{T}$. The true decision boundary is characterized by $\boldsymbol{w}_{\text{true}} = (0, 1, 0)^\mathsf{T}$. We denote the learned decision boundary as $\hat{\boldsymbol{w}}$. Next, we compute an error metric between the learned and true decision boundaries as follows. We first normalize the learned decision boundary and define $\hat{\boldsymbol{w}}_{\text{norm}} = \frac{\hat{\boldsymbol{w}}}{\|\hat{\boldsymbol{w}}\|_2}$. We do not need to do this for $\boldsymbol{w}_{\text{true}}$ since it is already normalized. Then, we use the error metric

$$\text{err}(\hat{\boldsymbol{w}}, \boldsymbol{w}_{\text{true}}) = \min\left(\|\hat{\boldsymbol{w}}_{\text{norm}} - \boldsymbol{w}_{\text{true}}\|_2, \|\hat{\boldsymbol{w}}_{\text{norm}} + \boldsymbol{w}_{\text{true}}\|_2\right). \tag{53}$$

Including both terms in the minimum is necessary due to unidentifiability of the signs of $\hat{\boldsymbol{w}}$.

Figure 5A compares this error metric across the 5 model fits for each vEM method. It is clear that the modified vEM approach consistently outperforms the standard vEM approach in terms of more accurately estimating the decision boundary. In addition, the error for standard vEM has much higher variance, since the algorithm is prone to getting stuck in local maxima of the ELBO. Figures 5B-C display the learned versus true decision boundaries in the latent space for two selected runs. We select the run from each vEM approach which achieved the lowest decision boundary error metric, as denoted by solid dots in fig. 5A. We find that the model fit with standard vEM learns a decision boundary which noticeably deviates from the true boundary. On the other hand, the model with with modified vEM recovers the true boundary almost perfectly. This illustrates that our modified approach dramatically improves kernel hyperparameter estimation in practice and enables the gpSLDS to be much more interpretable in the latent space.

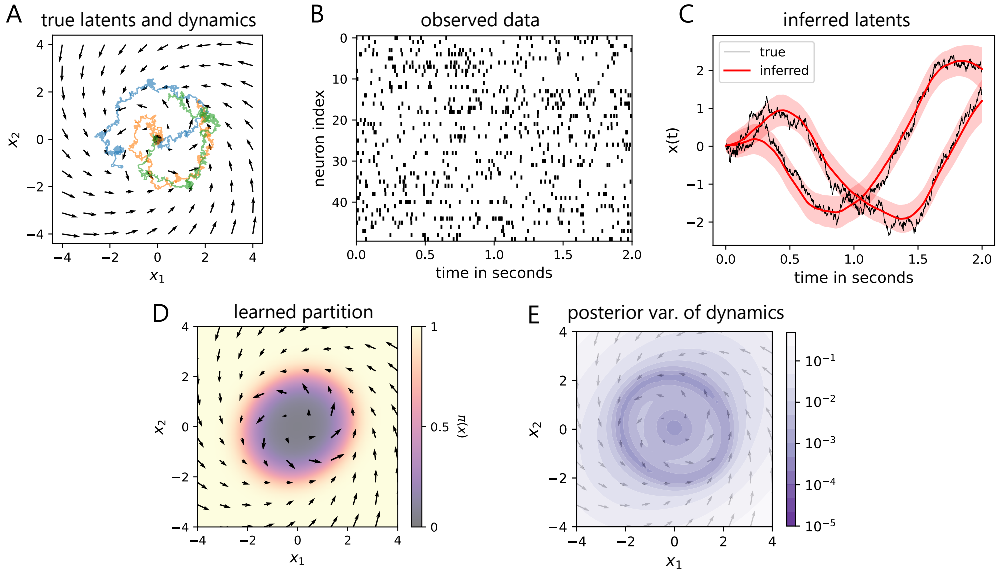


Figure 6: Additional synthetic data results on a 2D limit cycle from a gpSLDS fit with quadratic decision boundaries. **A.** True dynamics and true latent trajectories on 3 example trials used to generate the dataset. Dynamics are an unstable rotation and a stable rotation with fixed points at $(0, 0)$, separated by $x_1^2 + x_2^2 = 4$. **B.** Poisson process observations for an example trial. **C.** gpSLDS inferred latent trajectory with 95% posterior credible intervals for an example trial. **D.** The learned $\boldsymbol{\pi}(\boldsymbol{x})$ accurately recovers the true circular boundary between the two sets of linear dynamics. **E.** The gpSLDS learned posterior variance on dynamics. The posterior variance is low in regions heavily traversed by the true latent paths, and is high in regions with little to no data.

# D Additional synthetic data results

To further demonstrate the expressivity of the gpSLDS over the rSLDS, we apply the gpSLDS to a synthetic dataset where the true decision boundary between linear regimes is nonlinear. The rSLDS can only model linear decision boundaries in order for its inference algorithm to remain tractable.

For this example, we generate a synthetic dataset consisting of an unstable linear system and a stable linear system separated by the decision boundary $x_1^2 + x_2^2 = 4$. Both of the linear systems have fixed points at $(0, 0)$. The smooth combination of these linear systems results in a 2D limit cycle (fig. 6A). We simulate 30 trials of Poisson process observations from $D = 50$ neurons over $T = 2$ seconds (fig. 6B). We initialize the observation model parameters $\boldsymbol{C}$ and $\boldsymbol{d}$ using a Poisson LDS with data binned at 20ms. Then, we fit a gpSLDS with $J = 2$ regimes, and with $\boldsymbol{\pi}(\boldsymbol{x})$ modeled using the feature transformation

$$\boldsymbol{\phi}(\boldsymbol{x}) = \begin{bmatrix} 1 & \boldsymbol{x}_1^2 & \boldsymbol{x}_2^2 \end{bmatrix}^\intercal . \tag{54}$$

The results of this experiment are shown in fig. 6C-E. In fig. 6C we find that the gpSLDS successfully recovers the the true latent trajectory with accurate posterior credible intervals for an example trial. Furthermore, fig. 6D demonstrates that by using the quadratic feature transformation in eq. (54), the gpSLDS accurately learns the true flow field and true decision boundary $x_1^2 + x_2^2 = 4$. In addition, the values of $\boldsymbol{\pi}(\boldsymbol{x})$ smoothly transition between 0 and 1 near this boundary, highlighting the ability of our method to learn smooth dynamics if present. Lastly, in fig. 6E we plot the inferred posterior variance of our method. We find that the gpSLDS is more confident in regions of the latent space with more data (e.g. at the decision boundary), and less confident in regions of latent space with little to no data.

# E Computing resources

We fit all of our models on a NVIDIA A100 GPU on an internal computing cluster. A breakdown of approximate compute times for the main experiments in this paper includes:

- Synthetic data results in Section 4.1: 1.5 hours per model fit, $\sim$40 hours for the entire experiment.
- Real data results in Section 4.2: 1.5 hours per model fit, $\sim$8 hours for the entire experiment.
- Real data results in Section 4.3: 1 hour per model fit, $\sim$5 hours for the entire experiment.

We note that these estimates do not include the full set of experiments we performed while carrying out this project (such as preliminary or failed experiments).
